# Assessment of Out-of-Pocket Costs for Robotic Cancer Surgery in US Adults

**DOI:** 10.1001/jamanetworkopen.2019.19185

**Published:** 2020-01-15

**Authors:** Junaid Nabi, David F. Friedlander, Xi Chen, Alexander P. Cole, Jim C. Hu, Adam S. Kibel, Prokar Dasgupta, Quoc-Dien Trinh

**Affiliations:** 1 Division of Urology, Brigham and Women’s Hospital, Harvard Medical School, Boston, Massachusetts; 2 Center for Surgery and Public Health, Brigham and Women’s Hospital, Harvard Medical School, Boston, Massachusetts; 3 Department of Urology, Weill Cornell Medical College, New York, New York; 4 Medical Research Council Centre for Transplantation, National Institute for Health Research Biomedical Research Centre, King’s College, London, United Kingdom

## Abstract

**Importance:**

Expensive technologies—including robotic surgery—experience rapid adoption without evidence of superior outcomes. Although previous studies have examined perioperative outcomes and costs, differences in out-of-pocket costs for patients undergoing robotic surgery are not well understood.

**Objective:**

To assess out-of-pocket costs and total payments for 5 types of common oncologic procedures that can be performed using an open or robotic approach.

**Design, Setting, and Participants:**

A retrospective, cross-sectional, propensity score–weighted analysis was performed using deidentified insurance claims for 1.9 million enrollees from the MarketScan database from January 1, 2012, to December 31, 2017. The final study sample comprised 15 893 US adults aged 18 to 64 years who were enrolled in an employer-sponsored health plan. Patients underwent either an open or robotic radical prostatectomy, hysterectomy, partial colectomy, radical nephrectomy, or partial nephrectomy for a solid-organ malignant neoplasm. Statistical analysis was performed from December 18, 2018, to June 5, 2019.

**Exposures:**

Type of surgical procedure—robotic vs open.

**Main Outcomes and Measures:**

The primary outcome of interest was out-of-pocket costs associated with robotic and open surgery. The secondary outcome of interest was associated total payments.

**Results:**

Among 15 893 patients (11 102 men; mean [SD] age, 55.4 [6.6] years), 8260 underwent robotic and 7633 underwent open procedures; patients undergoing robotic hysterectomy were older than those undergoing open hysterectomy (mean [SD] age, 55.7 [6.7] vs 54.6 [7.2] years), and patients undergoing open radical nephrectomy had more comorbidities than those undergoing robotic radical nephrectomy (≥2 comorbidities, 658 of 861 [76.4%] vs 244 of 347 [70.3%]). After adjustment for baseline characteristics, the robotic approach was associated with lower out-of-pocket costs for all procedures: –$137.75 (95% CI, −$240.24 to −$38.63) for radical prostatectomy (*P* = .006); −$640.63 (95% CI, −$933.62 to −$368.79) for hysterectomy (*P* < .001); –$1140.54 (95% CI, −$1397.79 to −$896.54) for partial colectomy (*P* < .001); –$728.32 (95% CI, −$1126.90 to −$366.08) for radical nephrectomy (*P* < .001); and –$302.74 (95% CI, −$523.14 to −$97.10) for partial nephrectomy (*P* = .003). The robotic approach was similarly associated with lower adjusted total payments: –$3872.62 (95% CI, −$5385.49 to −$2399.04) for radical prostatectomy (*P* < .001); –$29 640.69 (95% CI, −$36 243.82 to −$23 465.94) for hysterectomy (*P* < .001); –$38 151.74 (95% CI, −$46 386.16 to −$30 346.22) for partial colectomy; (*P* < .001); –$33 394.15 (95% CI, −$42 603.03 to −$24 955.20) for radical nephrectomy (*P* < .001); and –$9162.52 (95% CI, −$12 728.33 to −$5781.99) for partial nephrectomy (*P* < .001).

**Conclusions and Relevance:**

This study found significant variation in perioperative costs according to surgical technique for both patients (out-of-pocket costs) and payers (total payments); the robotic approach was associated with lower out-of-pocket costs for all studied oncologic procedures.

## Key Points

**Question:**

Is undergoing robotic cancer surgery associated with lower out-of-pocket costs for patients compared with undergoing open surgery?

**Findings:**

This cross-sectional study of 15 893 patients undergoing open or robotic radical prostatectomy, hysterectomy, partial colectomy, radical nephrectomy, or partial nephrectomy found that robotic surgery was associated with lower out-of-pocket costs relative to open surgery for all oncologic procedures.

**Meaning:**

For the 5 procedures investigated, robotic cancer surgery may be more affordable for patients, highlighting an array of economic factors associated with the rapid adoption of this technology.

## Teaser

This cross-sectional study assesses out-of-pocket costs and total payments for 5 types of common oncologic procedures that can be performed using an open or robotic approach.

## Introduction

As of 2017, US national health expenditures stood at $3.5 trillion.^1^ Despite recent reforms aimed at containing increasing US health care expenditures, overall US health care spending remains on an unsustainable course.^2,3,4^ Consequently, renewed focus has been placed on the value of medical services rendered. Although value-driven initiatives in the United States have traditionally emphasized eliminating excessive administrative costs and/or physician reimbursements, the role of innovative—and costly—technologies such as robotic surgery in increased health care spending has not been well studied, to our knowledge.^5^ In the past 2 decades, an exponential increase in the adoption of minimally invasive surgery for the management of common malignant neoplasms has occurred.^6^ This adoption is due in part to early evidence of lower morbidity, hospital length of stay (LOS), and blood loss, as well as reduced postoperative analgesia requirements, associated with minimally invasive surgery.^7,8,9^ As a result, minimally invasive procedures have secured a more integral role in oncologic surgery. However, this change has occurred in the context of research demonstrating higher associated surgical costs and equivocal evidence of improved clinical outcomes.^10,11,12^

Most studies that have examined perioperative outcomes and costs associated with robotic surgery have been limited by a dearth of granular cost data, thereby precluding a systematic assessment of the true financial cost—and, by extension, value—associated with the rapid adoption of robotic surgery. The literature to date has focused on total health care spending associated with robotic surgery (usually estimated by using total charges), generally showing robotic surgery to be associated with higher mean direct hospital costs and lower health plan spending, and there has not been a comprehensive scientific inquiry into out-of-pocket (OOP) costs for patients, to our knowledge.^6,13^ To truly understand whether robotic surgery is beneficial compared with open surgery, it is important to capture all costs borne by the patient, not just those covered by payers. Furthermore, understanding the specific segment of patients affected by the costs of a particular procedure may help better elucidate the factors associated with growing health inequity.^14^ To examine this question, we used a large, nationally representative sample of patients to assess OOP costs and total payments for 5 types of common oncologic procedures that can be performed using an open or robotic approach.

## Methods

### Data Source

We queried the IBM Watson Health (formerly Truven Health Analytics) MarketScan Commercial Claims and Encounters database. As a Health Insurance Portability and Accountability Act–compliant database, it assembles information on insurance enrollment along with medical and drug claims for millions of individuals who receive health insurance coverage from their employers in the form of various health plans. The database captures unique information on inpatient, outpatient, and emergency department encounters, including OOP charges and claims on prescription drugs, using unique patient identifiers. We analyzed data collected from January 1, 2012, to December 31, 2017, which contained deidentified claims for 1.9 million enrollees, representing 260 employers, spread over 40 health plans with 350 unique carriers in the United States. This study followed the Strengthening the Reporting of Observational Studies in Epidemiology (STROBE) reporting guideline.^15^ As the data were deidentified, the study was deemed exempt by the Brigham and Women’s Hospital (Partners Healthcare) Institutional Review Board.

### Study Population

**Figure 1.**
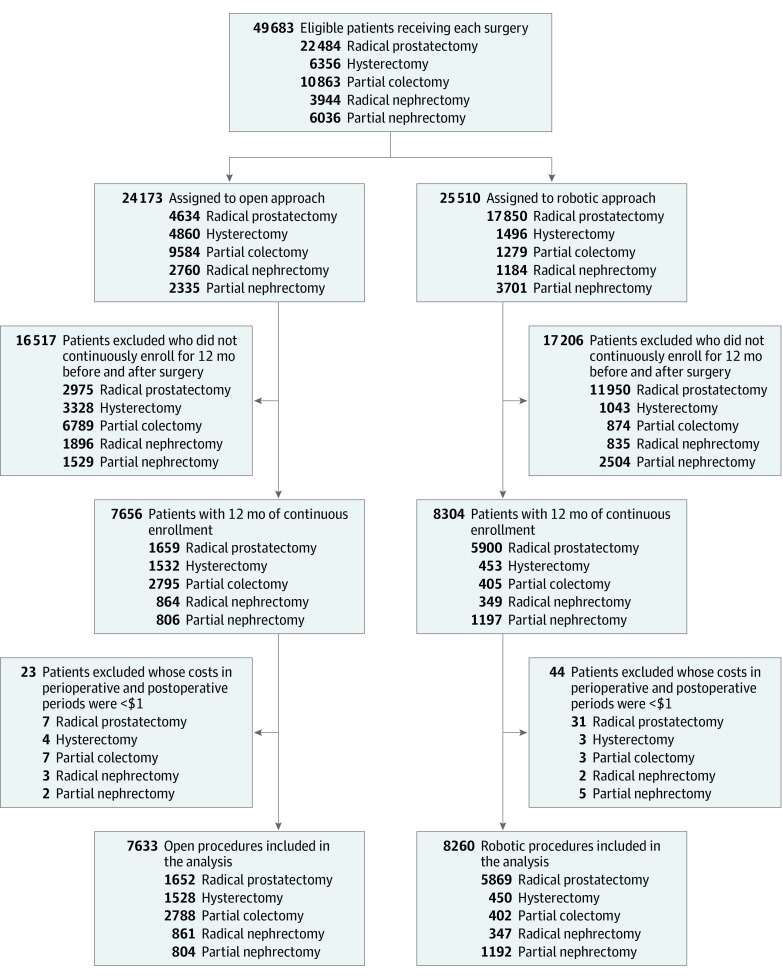
Flowchart of Patient and Procedure Selection in the Study

With a previously validated approach,^13^ we used *International Classification of Diseases, Ninth Revision* (*ICD-9*), *International Statistical Classification of Diseases and Related Health Problems, Tenth Revision* (*ICD-10*), and *Current Procedural Terminology* (*CPT*) codes to identify a sample of adults aged 18 to 64 years enrolled in an employer-sponsored health plan who underwent either an open or robotic radical prostatectomy (RP), hysterectomy (HYS), partial colectomy (PC), radical nephrectomy (RN), or partial nephrectomy (PN) for a solid-organ malignant neoplasm. For PC, we included colostomy and anterior resection. Adults older than 64 years were excluded because they are eligible for Medicare. Inclusion criteria were based on inpatient insurance claims for one of the aforementioned procedures between January 1, 2012, and December 31, 2017. To calculate the index surgical date, the earliest available date was considered, especially when multiple claims were available. Exclusion criteria included lack of 12 months of continuous insurance coverage in the same health plan before and after the index date, incurring a total expenditure of less than $1 (implying erroneous data collection), or incomplete demographic data (Figure 1).

### Outcomes

Our primary outcome of interest was OOP costs associated with robotic and open surgery; our secondary outcome of interest was total payments for patients who underwent 1 of the study’s 5 procedures. In accordance with previously published literature, we framed 3 time periods around the index surgical date: the baseline (−380 to −15 days), perioperative (−14 to 28 days), and postoperative (29 to 352 days) periods.^13,16^ Total payments associated with each surgical procedure were calculated by adding gross payments of all inpatient, outpatient, and pharmacy claims in the perioperative and postoperative periods (−14 to 352 days). Out-of-pocket costs were calculated by adding the coinsurance, copayment, and deductible of all inpatient, outpatient, and pharmacy claims during the perioperative and postoperative periods. We adjusted total payments to 2018 US dollars, relying on the general Consumer Price Index.

### Robotic Surgery

We used *ICD-9*, *ICD-10*, and *CPT* codes to identify the different types of open and laparoscopic surgery. We considered patients to be receiving robotic surgery if they had an open or laparoscopic surgery code, plus a robotic modifier (available in the eAppendix in the Supplement). Nonrobotic laparoscopic procedures were excluded from further analysis.^6,13^

### Statistical Analysis

Statistical analysis was performed from December 18, 2018, to June 5, 2019. Patient-level covariates included age, sex, Elixhauser comorbidity score, US Census region, urban vs rural residence, year of surgery, and health plan type (less restrictive vs more restrictive).^17^ Less restrictive plans included a comprehensive or preferred provider organization. More restrictive plans included an exclusive provider organization, a health maintenance organization, a noncapitated point-of-service, consumer-driven health plan, and a high-deductible health plan. The Elixhauser comorbidity score excluded the primary diagnosis for each cancer. To differentiate baseline characteristics between patients undergoing robotic surgery and patients undergoing open surgery, *t* tests were used for continuous variables and χ^2^ tests were used for categorical variables.

We first performed an inverse probability of treatment weighting (IPTW) propensity score analysis to address inherent differences in the covariates between the open and robotic surgery cohorts. We conducted IPTW propensity score analyses to balance the open vs robotic approach based on the patient-level covariates separately for each procedure cohort. Next, multivariable linear regression—weighted by the inverse of the probability of receiving robotic surgery based on baseline covariates and adjusting for baseline OOP or total payments—was used to estimate the independent association of surgical approach with OOP costs or total payments within the entire perioperative and postoperative period for each of the 5 procedure cohorts. The gamma distribution, which provides an accurate estimation of population means of health care costs,^18^ was used to report OOP costs and total payments.

In addition, we calculated IPTW-adjusted differences in hospital LOS for patients undergoing open or robotic procedures. Given that many of the robotic and minimally invasive procedures are increasingly performed on a short-stay basis, we wanted to understand the association of this status with the amount paid by the insurance company and to what extent longer hospital stays were associated with higher payments for open procedures.

We also conducted an outlier analysis for OOP costs and total payments between patients undergoing open surgery and patients undergoing robotic surgery separately for each procedure cohort, to examine the range of these expenses. This analysis was performed to address any observable variations in total payments and to examine whether these variations were artificially shifting the differences in mean payments between open and robotic procedures. In addition, because our main analysis examined the total payments associated with each surgical procedure in the entire perioperative and postoperative period (−14 to 352 days), to understand the broader overview of the association of the procedure with patient expenditures, we also wanted to understand the direct outcome in the shorter timeframe as reference points. Therefore, we conducted additional cost analyses for OOP and total payments at perioperative (−14 to 28 days) and 3-month (−14 to 90 days) periods.

All analyses were conducted using SAS, version 9.4 (SAS Institute Inc). A 2-tailed *P* < .05 was considered statistically significant.

## Results

### Baseline Demographic Characteristics

A total of 15 893 patients (11 102 men; mean [SD] age, 55.4 [6.6] years) underwent 1 of 5 surgical procedures: 8260 underwent robotic procedures, and 7633 underwent open procedures. From 2012 to 2017, there were 7521 patients who met inclusion criteria and underwent either open or robotic RP, 1208 patients who underwent either open or robotic RN, 1996 patients who underwent either open or robotic PN, 1978 patients who underwent either open or robotic HYS, and 3190 patients who underwent either open or robotic PC (Table 1). Robotic procedures represented 78.0% of the RP cohort (n = 5869), 28.7% of the RN cohort (n = 347), 59.7% of the PN cohort (n = 1192), 22.8% of the HYS cohort (n = 450), and 12.6% of the PC cohort (n = 402). In the RP cohort, both open (48.4%) and robotic (42.7%) procedures were observed in higher proportion in the South. Patients undergoing robotic HYS were older than those undergoing open HYS (mean [SD] age, 55.7 [6.7] vs 54.6 [7.2] years; *P* = .004). Patients undergoing open RN had more comorbidities than those undergoing robotic RN (≥2 comorbidities, 658 of 861 [76.4%] vs 244 of 347 [70.3%]; *P* = .01). Differences in baseline characteristics between patients undergoing open surgery and patients undergoing robotic surgery are described in Table 1.

**Table 1.** Baseline Demographic Characteristics of Patients Undergoing Open or Robotic Surgery in the MarketScan Database, 2012-2017

Characteristic	Radical Prostatectomy			Hysterectomy			Partial Colectomy			Radical Nephrectomy			Partial Nephrectomy		
	Open (n = 1652)	Robotic (n = 5869)	*P* Value	Open (n = 1528)	Robotic (n = 450)	*P* Value	Open (n = 2788)	Robotic (n = 402)	*P* Value	Open (n = 861)	Robotic (n = 347)	*P* Value	Open (n = 804)	Robotic (n = 1192)	*P* Value
Age, mean (SD), y	57.2 (4.8)	57.0 (4.7)	.14	54.6 (7.2)	55.7 (6.7)	.004	53.5 (7.6)	53.4 (7.0)	.73	53.8 (7.5)	54.3 (7.9)	.29	53.1 (8.1)	53.3 (8.1)	.63
Age group, No. (%)															
18-34	0	2 (0.03)	.16	21 (1.4)	9 (2.0)	<.001	57 (2.0)	6 (1.5)	.007	14 (1.6)	11 (3.2)	.24	23 (2.9)	35 (2.9)	.34
35-44	28 (1.7)	81 (1.4)		142 (9.3)	21 (4.7)		310 (11.1)	37 (9.2)		89 (10.3)	28 (8.1)		86 (10.7)	140 (11.7)	
45-54	384 (23.2)	1504 (25.6)		470 (30.8)	108 (24.0)		963 (34.5)	174 (43.3)		282 (32.8)	113 (32.6)		293 (36.4)	388 (32.6)	
55-64	1240 (75.1)	4282 (73.0)		895 (58.6)	312 (69.3)		1458 (52.3)	185 (46.0)		476 (55.3)	195 (56.2)		402 (50.0)	629 (52.8)	
Sex, No. (%)															
Male	1652 (100)	5869 (100)	NA	NA	NA	NA	1375 (49.3)	210 (52.2)	.27	532 (61.8)	229 (66.0)	.17	516 (64.2)	726 (60.9)	.13
Female	NA	NA		1528 (100)	450 (100)		1413 (50.7)	192 (47.8)		329 (38.2)	118 (34.0)		288 (35.8)	466 (39.1)	
Comorbidities, No. (%)															
0	313 (19.0)	1112 (19.0)	.94	151 (9.9)	44 (9.8)	.17	237 (8.5)	39 (9.7)	.26	64 (7.4)	43 (12.4)	.01	74 (9.2)	112 (9.4)	.91
1	507 (30.7)	1777 (30.3)		310 (20.3)	71 (15.8)		538 (19.3)	86 (21.4)		139 (16.1)	60 (17.3)		155 (19.3)	221 (18.5)	
≥2	832 (50.4)	2980 (50.8)		1067 (69.8)	335 (74.4)		2013 (72.2)	277 (68.9)		658 (76.4)	244 (70.3)		575 (71.5)	859 (72.1)	
Geographical region, No. (%)															
Northeast	281 (17.0)	998 (17.0)	<.001	320 (20.9)	95 (21.1)	<.001	497 (17.8)	107 (26.6)	<.001	111 (12.9)	51 (14.7)	<.001	210 (26.1)	274 (23.0)	<.001
North central	313 (19.0)	1475 (25.1)		371 (24.3)	108 (24.0)		626 (22.5)	80 (19.9)		172 (20.0)	101 (29.1)		140 (17.4)	300 (25.2)	
South	800 (48.4)	2506 (42.7)		602 (39.4)	143 (31.8)		1315 (47.2)	169 (42.0)		466 (54.1)	137 (39.5)		321 (39.9)	481 (40.4)	
West	229 (13.9)	810 (13.8)		218 (14.3)	100 (22.2)		314 (11.3)	45 (11.2)		101 (11.7)	56 (16.1)		116 (14.4)	129 (10.8)	
Unknown	29 (1.8)	80 (1.4)		17 (1.1)	4 (0.9)		36 (1.3)	1 (0.3)		11 (1.3)	2 (0.6)		17 (2.1)	8 (0.7)	
Residence, No. (%)															
Rural	343 (20.8)	943 (16.1)	<.001	235 (15.4)	53 (11.8)	.05	579 (20.8)	48 (11.9)	<.001	176 (20.4)	56 (16.1)	.08	127 (15.8)	175 (14.7)	.49
Urban	1309 (79.2)	4926 (83.9)		1293 (84.6)	397 (88.2)		2209 (79.2)	354 (88.1)		685 (79.6)	291 (83.9)		677 (84.2)	1017 (85.3)	
Health plan type, No. (%)															
Less restrictive^a^	1124 (68.0)	4000 (68.2)	.92	1019 (66.7)	296 (65.8)	.71	1877 (67.3)	270 (67.2)	.94	565 (65.6)	239 (68.9)	.27	542 (67.4)	825 (69.2)	.39
More restrictive^b^	528 (32.0)	1869 (32.0)		509 (33.3)	154 (34.2)		911 (32.7)	132 (32.8)		296 (34.4)	108 (31.1)		262 (32.6)	367 (30.8)	
Colostomy, No. (%)															
Yes	NA	NA	NA	NA	NA	NA	356 (12.8)	22 (5.5)	<.001	NA	NA	NA	NA	NA	NA
No	NA	NA		NA	NA		2432 (87.2)	380 (94.5)		NA	NA		NA	NA	
Low anterior resection, No. (%)															
Yes	NA	NA	NA	NA	NA	NA	593 (21.3)	152 (37.8)	<.001	NA	NA	NA	NA	NA	NA
No	NA	NA		NA	NA		2195 (78.7)	250 (62.2)		NA	NA		NA	NA	

Abbreviation: NA, not applicable.

^a^ Less restrictive health plans: comprehensive, preferred provider organization.

^b^ More restrictive health plans: basic or major medical, exclusive provider organization, health maintenance organization, noncapitated point of service, point of service with capitation or partially capitated point of service, consumer-driven health plan, and high-deductible health plan.

### OOP Costs

In IPTW-adjusted analyses accounting for the OOP costs in the baseline period (−380 to −15 days), the robotic approach was associated with lower OOP costs for all procedures examined: –$137.75 (95% CI, −$240.24 to −$38.63) for RP (*P* = .006); –$640.63 (95% CI, −$933.62 to −$368.79) for HYS (*P* < .001); –$1140.54 (95% CI, −$1397.79 to −$896.54) for PC (*P* < .001); –$728.32 (95% CI, −$1126.90 to −$366.08) for RN (*P* < .001); and –$302.74 (95% CI, −$523.14 to −$97.10) for PN (*P* = .003) (Table 2).

**Table 2.** Adjusted Differences in OOP Costs Between Patients Undergoing Open and Patients Undergoing Robotic Surgery

Surgery	Mean OOP Costs, $	Adjusted Difference in OOP, $ (95% CI)^a^	*P* Value
Radical prostatectomy			
Open	3151.43	137.75 (38.63-240.24)	.006
Robotic	2888.57		
Hysterectomy			
Open	3769.22	640.63 (368.79-933.62)	<.001
Robotic	3011.26		
Partial colectomy			
Open	4620.09	1140.54 (896.54-1397.79)	<.001
Robotic	3435.48		
Radical nephrectomy			
Open	4002.82	728.32 (366.08-1126.90)	<.001
Robotic	3371.95		
Partial nephrectomy			
Open	3177.02	302.74 (97.10-523.14)	.003
Robotic	2816.95		

Abbreviation: OOP, out-of-pocket.

^a^ Adjusted for OOP costs in the baseline period and weighted by the inverse probability of receiving robotic surgery based on baseline covariates.

### Total Payments

In IPTW-adjusted analyses accounting for the total payments in the baseline period (−380 to −15 days), the robotic approach was associated with lower total payments for all procedures examined: –$3872.62 (95% CI, −$5385.49 to −$2399.04) for RP (*P* < .001); –$29 640.69 (95% CI, −$36 243.82 to −$23 465.94) for HYS (*P* < .001); –$38 151.74 (95% CI, −$46 386.16 to −$30 346.22) for PC (*P* < .001); –$33 394.15 (95% CI, −$42 603.03 to −$24 955.20) for RN (*P* < .001); and –$9162.52 (95% CI, −$12 728.33 to −$5781.99) for PN (*P* < .001) (Table 3).

**Table 3.** Adjusted Differences in Total Costs Between Patients Undergoing Open and Patients Undergoing Robotic Surgery

Surgery	Total Costs, Mean, $	Adjusted Difference in Total Costs, $ (95% CI)^a^	*P* Value
Radical prostatectomy			
Open	54 529.42	3872.62 (2399.04-5385.49)	<.001
Robotic	49 406.32		
Hysterectomy			
Open	98 045.31	29 640.69 (23 465.94-36 243.82)	<.001
Robotic	68 503.97		
Partial colectomy			
Open	158 911.64	38 151.74 (30 346.22-46 386.16)	<.001
Robotic	113 033.10		
Radical nephrectomy			
Open	105 899.26	33 394.15 (24 955.20-42 603.03)	<.001
Robotic	77 434.54		
Partial nephrectomy			
Open	66 057.34	9162.52 (5781.99-12 728.33)	<.001
Robotic	55 791.82		

^a^ Adjusted for total costs in the baseline period and weighted by the inverse probability of receiving robotic surgery based on baseline covariates.

### Length of Stay

In IPTW-adjusted analyses, the robotic approach was associated with shorter LOS for all procedures examined: −0.94 days (95% CI, −1.02 to −0.85 days) for RP (*P* < .001); −2.28 days (95% CI, −2.53 to −2.04 days) for HYS (*P* < .001); −3.18 days (95% CI, −3.52 to −2.83 days) for PC (*P* < .001); −2.34 days (95% CI, −2.66 to −2.03 days) for RN (*P* < .001); and −1.59 days (95% CI, −1.77 to −1.41 days) for PN (*P* < .001) (eTable 1 in the Supplement).

### Additional Cost and Outlier Analysis

For the perioperative period (−14 to 28 days), adjusted OOP costs were significantly lower for the robotic option for PC (–$471.90 [95% CI, −$651.84 to −$305.81]; *P* < .001) and RN (–$570.46 [95% CI, −$855.66 to −$320.35]; *P* < .001) but not for RN, HYS, and PN (eTable 2A in the Supplement). In the same period, adjusted total payments were significantly lower for all robotic procedures except RP (eTable 2B in the Supplement). Last, at 3 months (−14 to 90 days), adjusted OOP costs as well as adjusted total payments were significantly lower for all robotic procedures except RP (eTable 3A and 3B in the Supplement). Our outlier analysis also demonstrated that, apart from infrequent values for OOP costs for RP, PC, RN, and PN and total payments for RP, HYS, PC, and RN, the variations remained generally narrow (eFigure in the Supplement).

## Discussion

**Figure 2.**
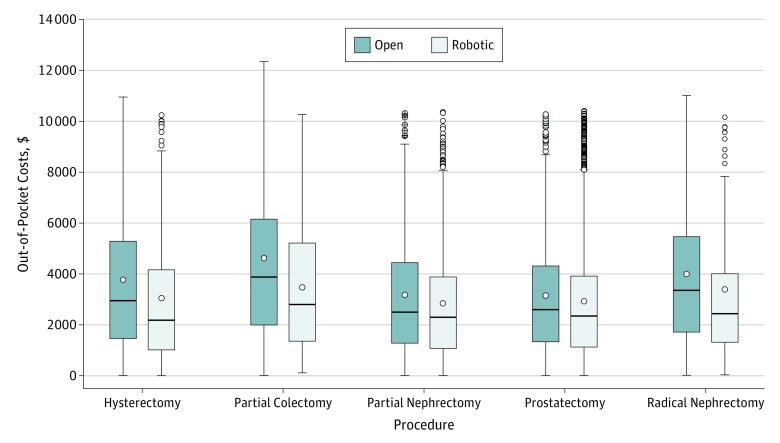
Differences in Out-of-Pocket Costs The circles outside the bars represent outlier values. The horizontal line within each box represents the median value. The circles inside the boxes represent the mean values of each group. The error bars below and above the boxes represent the minimum and maximum values, respectively.

In this study of 15 893 adults within a large nationally representative cohort of privately insured patients, we found significantly lower OOP and total payments associated with the robotic approach for all 5 studied oncologic procedures (Figure 2). Notwithstanding the equivocal evidence regarding clinical benefit^12^ and a contentious debate on the value rendered by robotic oncologic surgery, evidence suggests that the robotic approach is assuming a greater role in urologic, gynecologic, and general surgery procedures.^6,19^ This increase is in spite of evidence that patients express greater disillusionment with robotic surgery after the procedure,^20^ of gaps in the literature on long-term cost and quality-of-life implications for patients who may not benefit from robotic procedures, and of a recent US Food and Drug Administration warning against using the robotic approach in several cancer-related surgical procedures.^21^ Although previous investigations have focused on total health care spending associated with robotic surgery to determine the value of robotic surgery, it is necessary to understand whether the burden of these costs falls on the patients directly (in the form of higher OOP costs) or on the hospitals where patients seek care. This report provides the first comprehensive economic assessment, to our knowledge, of variations in total and OOP costs when comparing robotic and open surgery.

Our results of lower OOP and total payments for robotic surgery should be interpreted carefully, given the scope of our analysis. First, these analyses do not account for the costs of procuring and maintaining a robotic system (ranging from $0.5 to $2.5 million).^6^ Second, previous economic analyses have demonstrated that robotic surgery could be more expensive perioperatively than open surgery,^19,22^ when considering the costs of robotic maintenance, as well as disposable instruments (costs range from $600 to $1000, and the instruments are generally limited to 10 uses). Another factor that could be associated with these differences is hospital LOS, which has been demonstrated to be significantly reduced for patients undergoing robotic procedures.^9^ In our analysis, we found that the mean LOS was shorter for the robotic approach in all procedures examined. It is possible that these differences in LOS may also be a factor associated with higher payments for open surgery and may explain the differences in total payments, given that hospital-related costs likely exceed those of the other categories comprising total payments (eg, pharmaceuticals).

Our findings also contrast with those of previous studies. Yu et al^23^ reported an approximately $1000 additional cost for those undergoing robotic prostatectomy. However, their analysis relied on inpatient costs for all payers, whereas our analysis examines perioperative costs extending beyond the inpatient stay, for private payers. Also, Nguyen et al^24^ found that minimally invasive prostatectomy (mostly robotic assisted) costs $236 more than open prostatectomy. Their analysis included older patients with Medicare coverage and calculated costs over the course of a year, which would understandably include health care use not observed among younger, privately insured patients (the population of our study). In our outlier analysis, we observed that, apart from occasional values for OOP costs for RP, PC, RN, and PN and total payments for RP, HYS, PC, and RN, the variations generally remained narrow. These outlier values, however, could be a result of patient-level differences or other factors. However, these outlier values are unlikely to shift the differences in mean payments between open and robotic procedures, and because we have accounted for patient-level baseline characteristics, we decided to include those values in our analysis.

Additional cost calculations demonstrated that, with the increase in duration of care, the differences in costs became increasingly pronounced. For the perioperative periods (−14 to 28 days), adjusted OOP costs were significantly lower for the robotic option for PC and RN but not for RN, HYS, and PN. In the same period, adjusted total payments were significantly lower for all robotic procedures except RP. At 3 months (−14 to 90 days), adjusted OOP costs as well as adjusted total payments were significantly lower for all robotic procedures except RP.

Our analyses indicate that the additional costs of robot acquisition and maintenance are seemingly not paid by private health insurers (approximated here by total payments) or patients (approximated here by OOP costs); if such is the case, by extension, these costs appear to be absorbed by the hospitals. Although the exact reasons why hospitals have been willing to absorb or subsidize costs associated with robotic surgery remain unclear, there are some plausible explanations. In recent years, there has been a rapid diffusion in the adoption of robotic surgery.^25^ Given this trend, it is possible that the marginal cost of undergoing robotic vs open surgery is lower—that is, while the total reimbursements are lower for the hospitals, the net profit is still higher. Our analyses of the most recent years available (2012-2017) provide a more thorough understanding of this trend because previous investigations relied on data from the last decade.^13^

Since the last decade, the adoption of robotic surgery has increased considerably.^25^ Although profitability remains an important motivator for rapidly adopting robotic surgery,^26^ a key reason why hospitals are willing to absorb the high upfront costs of robotic surgery is patient demand.^27^ Evidence supports the finding that direct-to-consumer advertising of robotic surgery increases demand.^28,29^ This higher demand could influence hospitals offering robotic surgery as a viable option to retain market share and stay competitive. Our finding of significantly lower OOP costs associated with the robotic approach is likely to compound this trend. In addition to acquiescing to patient demand, another mechanism that may be associated with the rapid acquisition and implementation of robotic surgery could be the ability of nonprofit hospitals to access tax-exempt financial instruments toward using debt for operational growth.^30^

The adoption of robotic surgery has coincided with the centralization of surgical procedures, most notably for RP in the United States. Investigators have shown that institutions acquiring surgical robotics have seen a dramatic increase in their surgical volume. Specifically, Riikonen et al^31^ demonstrated that the principal outcome of national-level adoption of robotic surgery in Finland led to the immediate and unpremeditated centralization for prostate cancer surgery. It has also been reported that high-volume surgeons in the United States at teaching and large hospitals have swiftly adopted the robotic approach.^32^ Evidence also shows that regionalization has been observed at a higher rate for the robotic approach compared with the open approach.^33^ In the United Kingdom, Aggarwal et al^27^ showed that competitive factors and centralization of services have led to greater investment in building cancer surgery units that use robotic surgery as a primary treatment modality. Several studies have shown the conditions in which the excess costs of robotic surgery are mitigated: when hospital volume is high and operative time is low, robotic surgery can cost less.^34,35,36^

Furthermore, another reason why hospitals may be willing to absorb the costs of the acquisition of surgical robotics could be a consequence of changes in residency training programs. Recent evidence points to surgical residency programs increasingly training their residents in operating on robotic platforms,^37^ leading trainees to be better equipped for the robotic approach. It has also been suggested that the laparoscopic approach has a steep learning curve, which could further augment training on a robotic platform.^38^ This change is compounded by reports that high-volume centers are more likely to use a robotic platform.^39^ Because teaching hospitals tend to be high-volume centers, it is possible there is an increased focus—albeit unintended—on learning common surgical procedures on a robotic platform, especially in urology and gynecology. Residency training programs and hospitals should be cautious about this trend because it may be associated with long-term harmful consequences, such as the development of surgeons who are not well trained in open procedures, which may impede timely access to surgical care in low-resource settings that have yet to acquire robotic surgical platforms.

Given that the cost of the acquisition and maintenance of surgical robotics are not accounted for in this analysis, it is plausible that robotic surgery exhibits small gains compared with the conventional open approaches through shorter LOS, use of pain medication, and use of laboratory tests, among other factors. From an accounting perspective, total cost differences between robotic and open approaches could also be associated with fewer postacute care days and lower morbidity.^7,8,13^ Our findings are concordant with a recent investigation by Motz et al,^40^ who found that transoral robotic surgery was associated with significantly lower total treatment-related costs (–$22 724). Previous economic analyses have explored the possibility that inclusion of robotic surgery has discrete benefits for hospitals in terms of revenue because the costs of procuring and maintaining a robotic system (ranging from $0.5 to $2.5 million) can be recuperated when a steady volume of 100 to 150 procedures per year is maintained.^35,41^

### Limitations

Although our analysis accounted for potential confounders, our study has certain limitations. The MarketScan database does not include details on patient race/ethnicity and clinical factors such as stage of cancer, grade of cancer, preceding abdominal surgical procedures, or body mass index, factors that could influence the decision to undergo open or robotic surgery. As such, clinically meaningful differences among patients undergoing these procedures may not be satisfactorily captured; while we have adjusted for baseline characteristics, it is possible that omitted-variable bias could affect our analysis. Also, administrative data are limited in their ability to control for unknown confounders that could explicate these differences. Our analysis is limited by calculating medical expenditure data for employees of self-insured firms alone. Although it is unlikely that this limitation would be associated with the type of procedure that a given patient would undergo, it could limit the generalizability of our analysis. Another limitation could be the exclusion of Medicare beneficiaries, who are covered under a different reimbursement structure. However, OOP costs and total payments are much more relevant for those covered through private insurance because this type of plan tends to have a tier-based structure that can greatly limit the ability and affordability of patients to choose between robotic and open surgery options. We also recognize the absence of measurement and comparison of clinical outcomes under the open and robotic approach. This limitation was mitigated by undertaking an assessment of 5 different types of procedures to allow for variability because each of these procedures has previously reported similar clinical outcomes between the open and robotic options.

## Conclusions

We observed significant variation in perioperative costs according to surgical technique for both patients (OOP costs) and payers (total payments), with the robotic approach associated with significantly lower OOP costs for all studied oncologic procedures. These results highlight the complexity of economic factors that are associated with the rapid adoption and possible subsidization of the robotic approach for common surgically amenable conditions and lay a foundation for future work on this issue.

## Notes

Supplement. **eFigure.** Outlier Analysis for Out-of-Pocket (OOP) Costs and Total Payments Between Patients Undergoing Open and Robotic Radical Prostatectomy, Hysterectomy, Partial Colectomy, Radical Nephrectomy, and Partial Nephrectomy **eTable 1.** Adjusted Differences in Length of Stay (LoS) for Patients Undergoing Open and Robotic Radical Prostatectomy, Hysterectomy, Partial Colectomy, Radical Nephrectomy, and Partial Nephrectomy – Weighted by the Inverse Probability of Receiving Robotic Surgery Based on Baseline Covariates **eTable 2.** Adjusted Differences in Perioperative (-14 to +28 days) Out-of-Pocket Costs and Total Payments for Patients Undergoing Open and Robotic Radical Prostatectomy, Hysterectomy, Partial Colectomy, Radical Nephrectomy, and Partial Nephrectomy – Adjusted for OOP Costs in Baseline Period and Weighted by the Inverse Probability of Receiving Robotic Surgery Based on Baseline Covariates **eTable 3.** Adjusted Differences in 3 Month (-14 to +90 days) Out-of-Pocket Costs and Total Payments for Patients Undergoing Open and Robotic Radical Prostatectomy, Hysterectomy, Partial Colectomy, Radical Nephrectomy, and Partial Nephrectomy – Adjusted for OOP Costs in Baseline Period and Weighted by the Inverse Probability of Receiving Robotic Surgery Based on Baseline Covariates **eAppendix.** *ICD-9*, *ICD-10*, and *CPT* Codes for Disease States and Procedures Used in this Analysis
