# Supplementary material for: Assessment of Out-of-Pocket Costs for Robotic Cancer Surgery in US Adults
Source: JAMA Netw Open. 2020 Jan 15;3(1):e1919185. doi: 10.1001/jamanetworkopen.2019.19185 (PMC6991257; doi:10.1001/jamanetworkopen.2019.19185)
Supplement: Supplement. — eFigure. Outlier Analysis for Out-of-Pocket (OOP) Costs and Total Payments Between Patients Undergoing Open and Robotic Radical Prostatectomy, Hysterectomy, Partial Colectomy, Radical Nephrectomy, and Partial Nephrectomy eTable 1. Adjusted Differences in Length of Stay (LoS) for Patients Undergoing Open and Robotic Radical Prostatectomy, Hysterectomy, Partial Colectomy, Radical Nephrectomy, and Partial Nephrectomy – Weighted by the Inverse Probability of Receiving Robotic Surgery Based on Baseline Covariates eTable 2. Adjusted Differences in Perioperative (-14 to +28 days) Out-of-Pocket Costs and Total Payments for Patients Undergoing Open and Robotic Radical Prostatectomy, Hysterectomy, Partial Colectomy, Radical Nephrectomy, and Partial Nephrectomy – Adjusted for OOP Costs in Baseline Period and Weighted by the Inverse Probability of Receiving Robotic Surgery Based on Baseline Covariates eTable 3. Adjusted Differences in 3 Month (-14 to +90 days) Out-of-Pocket Costs and Total Payments for Patients Undergoing Open and Robotic Radical Prostatectomy, Hysterectomy, Partial Colectomy, Radical Nephrectomy, and Partial Nephrectomy – Adjusted for OOP Costs in Baseline Period and Weighted by the Inverse Probability of Receiving Robotic Surgery Based on Baseline Covariates eAppendix. ICD-9, ICD-10, and CPT Codes for Disease States and Procedures Used in this Analysis [file jamanetwopen-3-e1919185-s001.pdf]

Supplementary Online Content

Nabi J, Friedlander DF, Chen X, et al. Assessment of out-of-pocket costs for robotic cancer surgery in US adults. *JAMA Netw Open.* 2020;3(1):e1919185. doi:10.1001/jamanetworkopen.2019.19185

eFigure. Outlier Analysis for Out-of-Pocket (OOP) Costs and Total Payments Between Patients Undergoing Open and Robotic Radical Prostatectomy, Hysterectomy, Partial Colectomy, Radical Nephrectomy, and Partial Nephrectomy

eTable 1. Adjusted Differences in Length of Stay (LoS) for Patients Undergoing Open and Robotic Radical Prostatectomy, Hysterectomy, Partial Colectomy, Radical Nephrectomy, and Partial Nephrectomy – Weighted by the Inverse Probability of Receiving Robotic Surgery Based on Baseline Covariates

eTable 2. Adjusted Differences in Perioperative (-14 to +28 days) Out-of-Pocket Costs and Total Payments for Patients Undergoing Open and Robotic Radical Prostatectomy, Hysterectomy, Partial Colectomy, Radical Nephrectomy, and Partial Nephrectomy – Adjusted for OOP Costs in Baseline Period and Weighted by the Inverse Probability of Receiving Robotic Surgery Based on Baseline Covariates

eTable 3. Adjusted Differences in 3 Month (-14 to +90 days) Out-of-Pocket Costs and Total Payments for Patients Undergoing Open and Robotic Radical Prostatectomy, Hysterectomy, Partial Colectomy, Radical Nephrectomy, and Partial Nephrectomy – Adjusted for OOP Costs in Baseline Period and Weighted by the Inverse Probability of Receiving Robotic Surgery Based on Baseline Covariates

eAppendix. *ICD-9*, *ICD-10*, and *CPT* Codes for Disease States and Procedures Used in this Analysis

This supplementary material has been provided by the authors to give readers additional information about their work.

© 2020 Nabi J et al. *JAMA Network Open.*

eFigure. Outlier Analysis for Out-of-Pocket (OOP) Costs and Total Payments Between Patients Undergoing Open and Robotic Radical Prostatectomy, Hysterectomy, Partial Colectomy, Radical Nephrectomy, and Partial Nephrectomy

Prostatectomy

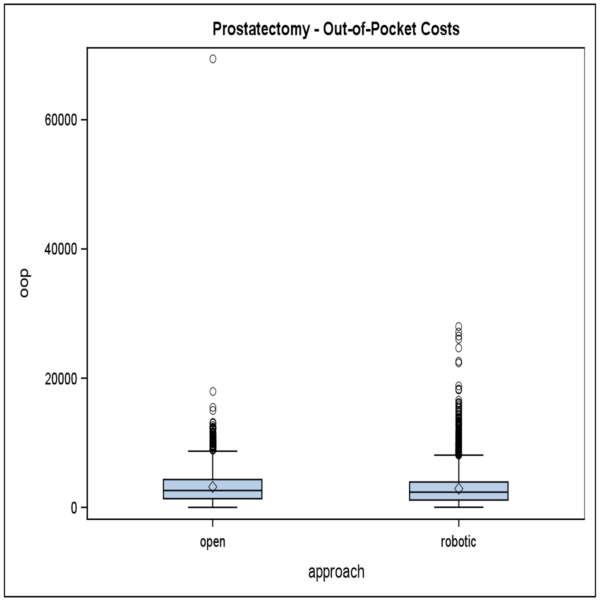

© 2020 Nabi J et al. *JAMA Network Open.*

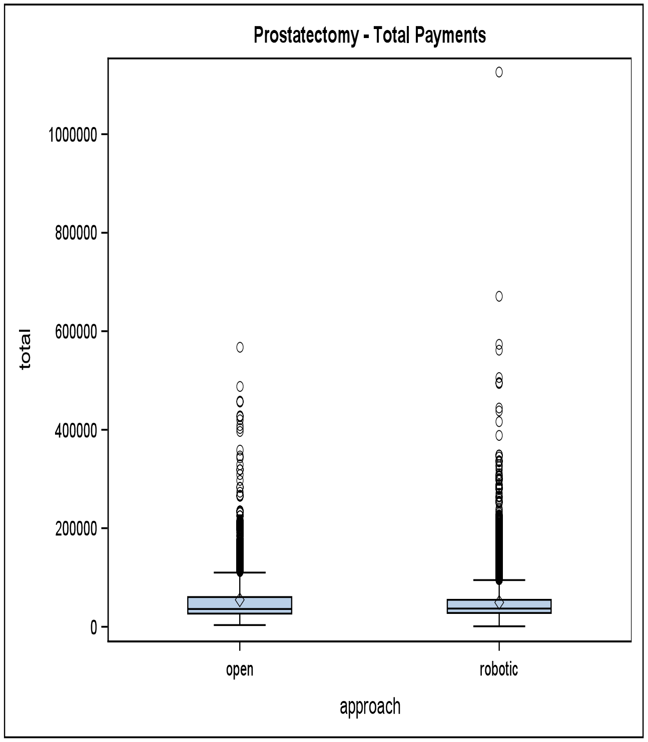

© 2020 Nabi J et al. *JAMA Network Open.*

Hysterectomy

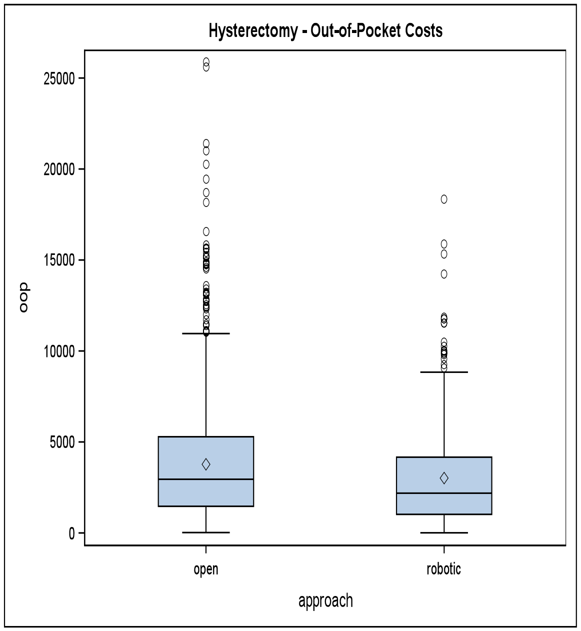

© 2020 Nabi J et al. *JAMA Network Open.*

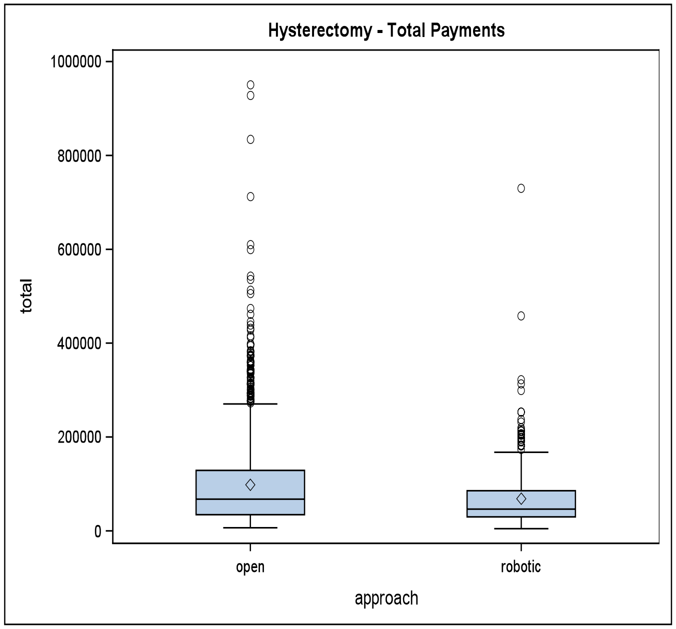

© 2020 Nabi J et al. *JAMA Network Open.*

Partial colectomy

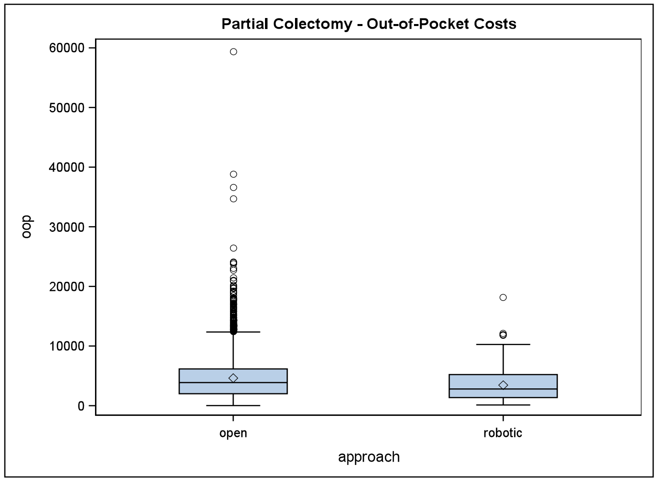

© 2020 Nabi J et al. *JAMA Network Open.*

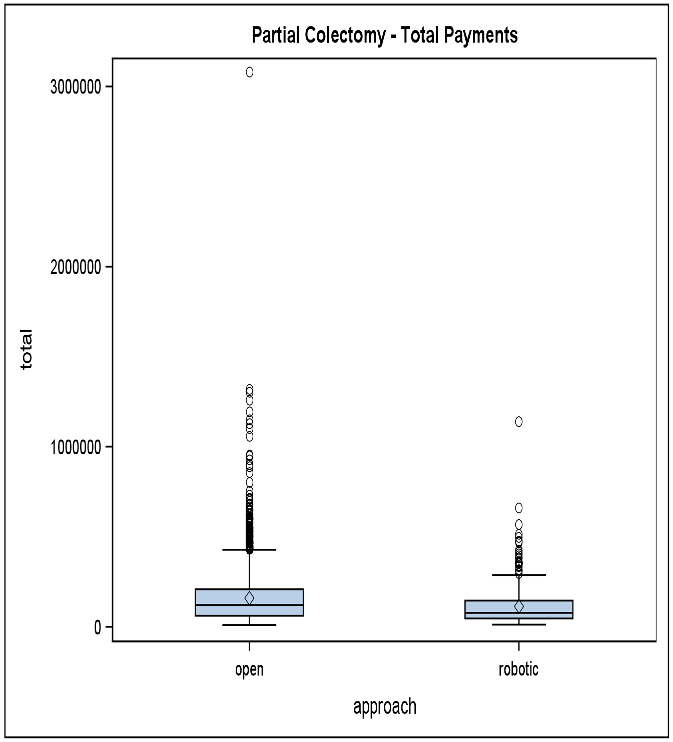

© 2020 Nabi J et al. *JAMA Network Open.*

Radical nephrectomy

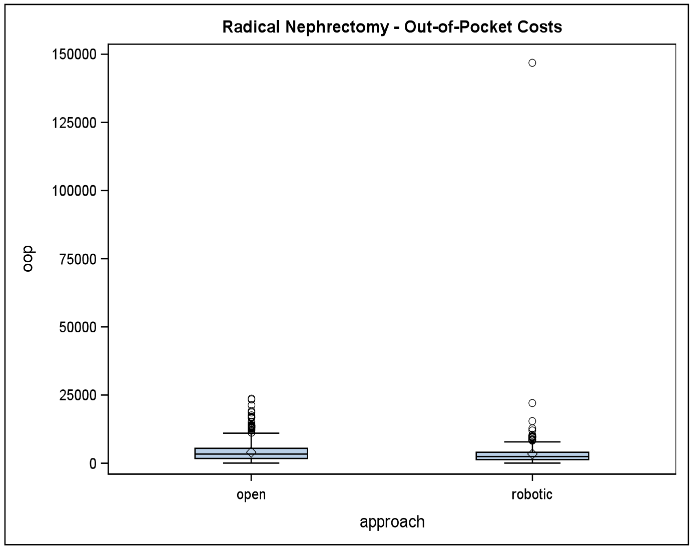

© 2020 Nabi J et al. *JAMA Network Open.*

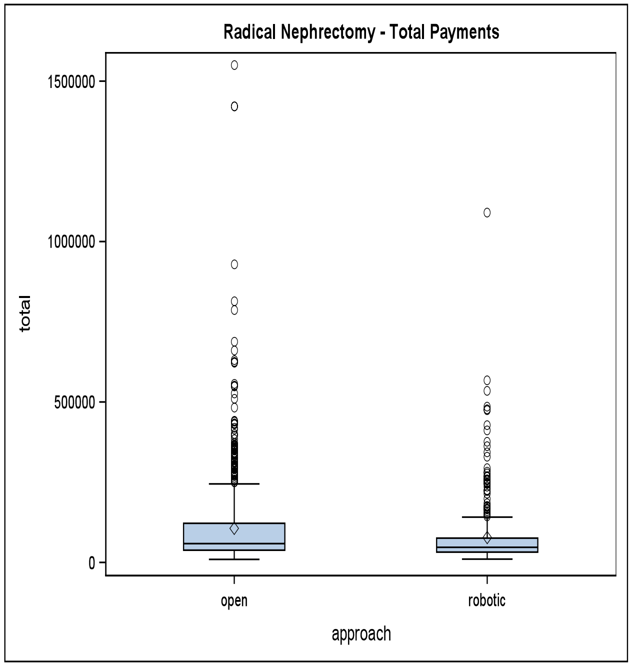

© 2020 Nabi J et al. *JAMA Network Open.*

Partial nephrectomy

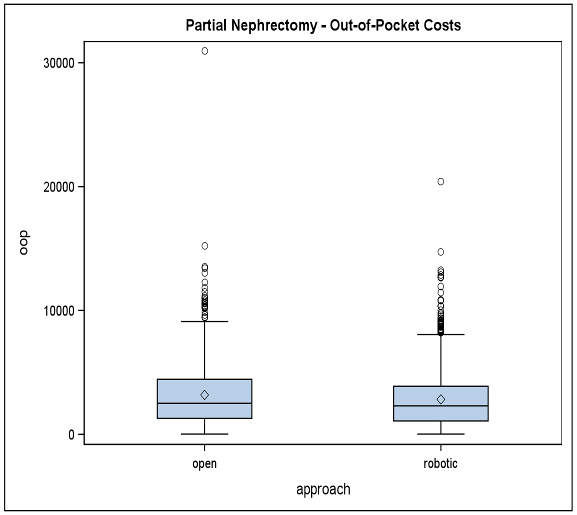

© 2020 Nabi J et al. *JAMA Network Open.*

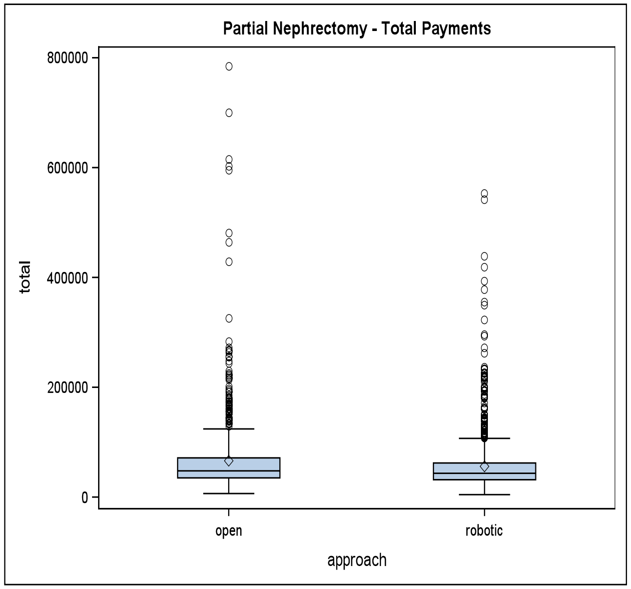

© 2020 Nabi J et al. *JAMA Network Open.*

eTable 1. Adjusted Differences in Length of Stay (LoS) for Patients Undergoing Open and Robotic Radical Prostatectomy, Hysterectomy, Partial Colectomy, Radical Nephrectomy, and Partial Nephrectomy – Weighted by the Inverse Probability of Receiving Robotic Surgery Based on Baseline Covariates

Open vs. Robotic – Length of Stay				
Surgery		**LOS (Mean)**	**Adjusted Difference in LOS (95% CI)**	***P* Value**
Radical Prostatectomy	Open	2.47	0.94 (0.85 to 1.02)	**<.001**
	Robotic	1.57		
Hysterectomy	Open	3.95	2.28 (2.04 to 2.53)	**<.001**
	Robotic	1.66		
Partial Colectomy	Open	7.72	3.18 (2.83 to 3.52)	**<.001**
	Robotic	4.32		
Radical Nephrectomy	Open	4.87	2.34 (2.03 to 2.66)	**<.001**
	Robotic	2.49		
Partial Nephrectomy	Open	3.96	1.59 (1.41 to 1.77)	**<.001**
	Robotic	2.38		

© 2020 Nabi J et al. *JAMA Network Open.*

eTable 2. Adjusted Differences in Perioperative (-14 to +28 days) Out-of-Pocket Costs and Total Payments for Patients Undergoing Open and Robotic Radical Prostatectomy, Hysterectomy, Partial Colectomy, Radical Nephrectomy, and Partial Nephrectomy – Adjusted for OOP Costs in Baseline Period and Weighted by the Inverse Probability of Receiving Robotic Surgery Based on Baseline Covariates

eTable 2A. Adjusted Differences in Perioperative (-14 to +28 days) Out-of-Pocket Costs for Patients Undergoing Open and Robotic Radical Prostatectomy, Hysterectomy, Partial Colectomy, Radical Nephrectomy, and Partial Nephrectomy – Adjusted for OOP Costs in Baseline Period and Weighted by the Inverse Probability of Receiving Robotic Surgery Based on Baseline Covariates

Open vs. Robotic – Perioperative Out-of-pocket (OOP) Costs (-14 to +28 days)				
Surgery		**OOP (Mean)**	**Adjusted Difference in OOP (95% CI)**	***P* Value**
Radical Prostatectomy	Open	1924.67	34.17 (-54.12 to 127.09)	0.45
	Robotic	1822.53		
Hysterectomy	Open	1928.09	93.26 (-93.06 to 299.65)	0.33
	Robotic	1739.91		
Partial Colectomy	Open	2168.83	471.90 (305.81 to 651.84)	**<.001**
	Robotic	1709.63		
Radical Nephrectomy	Open	2037.52	570.46 (320.35 to 855.66)	**<.001**
	Robotic	1436.99		
Partial Nephrectomy	Open	1666.66	121.52 (-43.32 to 304.96)	0.15
	Robotic	1528.62		

© 2020 Nabi J et al. *JAMA Network Open.*

eTable 2B. Adjusted Differences in Perioperative (-14 to +28 days) Total Payments for Patients Undergoing Open and Robotic Radical Prostatectomy, Hysterectomy, Partial Colectomy, Radical Nephrectomy, and Partial Nephrectomy – Adjusted for OOP Costs in Baseline Period and Weighted by the Inverse Probability of Receiving Robotic Surgery Based on Baseline Covariates

Open vs. Robotic – Perioperative Total Payment (-14 to +28 days)				
Surgery		**Total Payment (Mean)**	**Adjusted Difference in Total Payment (95% CI)**	***P* Value**
Radical Prostatectomy	Open	31924.78	-529.14 (-1234.95 to 189.94)	0.15
	Robotic	32169.64		
Hysterectomy	Open	40322.73	6145.89 (4235.13 to 8151.60)	**<.001**
	Robotic	34515.97		
Partial Colectomy	Open	61321.08	10168.08 (7727.05 to 12711.52)	**<.001**
	Robotic	49508.91		
Radical Nephrectomy	Open	44289.99	7572.60 (5055.35 to 10241.77)	**<.001**
	Robotic	37774.34		
Partial Nephrectomy	Open	40363.91	3611.88 (1811.26 to 5497.84)	**<.001**
	Robotic	36151.54		

© 2020 Nabi J et al. *JAMA Network Open.*

eTable 3. Adjusted Differences in 3 Month (-14 to +90 days) Out-of-Pocket Costs and Total Payments for Patients Undergoing Open and Robotic Radical Prostatectomy, Hysterectomy, Partial Colectomy, Radical Nephrectomy, and Partial Nephrectomy – Adjusted for OOP Costs in Baseline Period and Weighted by the Inverse Probability of Receiving Robotic Surgery Based on Baseline Covariates

eTable 3A. Adjusted Differences in 3 Month (-14 to +90 days) Out-of-Pocket Costs for Patients Undergoing Open and Robotic Radical Prostatectomy, Hysterectomy, Partial Colectomy, Radical Nephrectomy, and Partial Nephrectomy – Adjusted for OOP Costs in Baseline Period and Weighted by the Inverse Probability of Receiving Robotic Surgery Based on Baseline Covariates

Open vs. Robotic – Out-of-pocket (OOP) Costs (-14 to +90 days)				
Surgery		**OOP (Mean)**	**Adjusted Difference in OOP (95% CI)**	***P* Value**
Radical Prostatectomy	Open	2091.01	64.82 (-21.33 to 154.91)	0.14
	Robotic	1942.93		
Hysterectomy	Open	2388.69	317.70 (105.98 to 550.10)	**0.002**
	Robotic	1981.38		
Partial Colectomy	Open	2721.33	601.98 (418.89 to 798.63)	**<.001**
	Robotic	2104.63		
Radical Nephrectomy	Open	2356.11	685.24 (425.02 to 978.33)	**<.001**
	Robotic	1626.15		
Partial Nephrectomy	Open	1886.20	185.16 (18.13 to 368.99)	**0.028**
	Robotic	1673.47		

© 2020 Nabi J et al. *JAMA Network Open.*

eTable 3B. Adjusted Differences in 3 Month (-14 to +90 days) Total Payments for Patients Undergoing Open and Robotic Radical Prostatectomy, Hysterectomy, Partial Colectomy, Radical Nephrectomy, and Partial Nephrectomy – Adjusted for OOP Costs in Baseline Period and Weighted by the Inverse Probability of Receiving Robotic Surgery Based on Baseline Covariates

Open vs. Robotic – Total Payment (-14 to +90 days)				
Surgery		**Total Payment (Mean)**	**Adjusted Difference in Total Payment (95% CI)**	***P* Value**
Radical Prostatectomy	Open	35663.54	644.14 (-159.86 to 1469.57)	0.11
	Robotic	34461.59		
Hysterectomy	Open	59142.16	13224.77 (10059.79 to 16562.46)	**<.001**
	Robotic	46436.41		
Partial Colectomy	Open	86452.70	16418.83 (12844.47 to 20149.88)	**<.001**
	Robotic	67241.19		
Radical Nephrectomy	Open	55759.75	11979.61 (8404.30 to 15797.55)	**<.001**
	Robotic	45098.97		
Partial Nephrectomy	Open	46725.43	6257.15 (4050.93 to 8569.35)	**<.001**
	Robotic	39578.69		

© 2020 Nabi J et al. *JAMA Network Open.*

eAppendix. *ICD-9*, *ICD-10,* and *CPT* Codes for Disease States and Procedures Used in this Analysis

Category	*ICD-9* – DX	*ICD-10* – DX	Procedure	*ICD-9* – Proc	*ICD-10* – Proc	*CPT*
**Prostate Cancer**	185	C61	**Open Radical Prostatectomy**	60.5	0VT00ZZ, 0VT07ZZ	55840, 55842, 55845
			**Laparoscopic Radical Prostatectomy**	N/A	0VT04ZZ, 0VT08ZZ	55866
			**Robotic Assisted Laparoscopic Prostatectomy**	Above + 17.4, 17.41, 17.42, 17.43, 17.44, 17.45, 17.49	Above + 8E0W4CZ, 8E0W7CZ, 8E0WXCZ, 8E0W0CZ, 8E0W3CZ, 8E0W8CZ	Above + S2900 (HCPCS)
**Cervical and Endometrial/Uterine Cancer**	179, 182, 182.0, 182.1, 182.8	C55, C54, C54.0, C54.1, C54.2, C54.3, C54.8, C54.9	**Open Hysterectomy**	Total: 68.4, 68.49, 68.6, 68.69 Subtotal: 68.3, 68.39	Total: 0UT90ZZ + 0UTC0ZZ Subtotal: 0UT90ZL	Total: 58150, 58152, 58200, 58210 Subtotal: 58180
			**Laparoscopic Hysterectomy**	Total:	Total: 0UT94ZZ + 0UTC4ZZ	Total:

© 2020 Nabi J et al. *JAMA Network Open.*

				68.41, 68.61<br>Subtotal:<br>68.31<br>Vaginal:<br>68.5, 68.51, 68.59, 68.7, 68.71, 68.79	Subtotal: 0UT94ZL<br><br>Vaginal: 0UT9FZL, 0UT9FZZ + 0UTC4ZZ, 0UT97ZL, 0UT98ZL, 0UT97ZZ or 0UT98ZZ + 0UTC7ZZ or 0UTC8ZZ	58548, 58570, 58571, 58572, 58573, 58575<br>Subtotal:<br>58541, 58542, 58543, 58544<br>Vaginal:<br>58260, 58267, 58262, 58263, 58270, 58290, 58293, 58291, 58292, 58294, 58285, 58275, 58280, 58550, 58552,

© 2020 Nabi J et al. *JAMA Network Open.*

						58553, 58554
			**Robotic Assisted Hysterectomy**	Above + 17.4, 17.41, 17.42, 17.43, 17.44, 17.45, 17.49	Above + 8E0W4CZ, 8E0W7CZ, 8E0WXCZ, 8E0W0CZ, 8E0W3CZ, 8E0W8CZ	Above + S2900 (HCPCS)
**Colon Cancer**	153, 153.0, 153.1, 153.2, 153.3, 153.4, 153.5, 153.6, 153.7, 153.8, 153.9	C18, C18.0, C18.1, C18.2, C18.3, C18.4, C18.5, C18.6, C18.7, C18.8, C18.9	**Open Partial Colectomy**	45.41, 45.71, 45.72, 45.73, 45.74, 45.75, 45.76, 45.79, 45.7	0DTH0ZZ, 0DTH7ZZ, 0DTK0ZZ, 0DTK7ZZ, 0DTF0ZZ, 0DTF7ZZ, 0DTL0ZZ, 0DTL7ZZ, 0DTM0ZZ, 0DTM7ZZ, 0DTG0ZZ, 0DTG7ZZ, 0DTN0ZZ, 0DTN7ZZ, 0DBE0ZZ, 0DBE7ZZ, 0DBH0ZZ, 0DBH7ZZ, 0DBK0ZZ, 0DBK7ZZ, 0DBF0ZZ, 0DBF7ZZ,	44140, 44141, 44143, 44144, 44145, 44146, 44147, 44160

© 2020 Nabi J et al. *JAMA Network Open.*

					0DBL0ZZ,<br>0DBL7ZZ,<br>0DBM0ZZ,<br>0DBM7ZZ,<br>0DBG0ZZ,<br>0DBG7ZZ,<br>0DBN0ZZ,<br>0DBN7ZZ	
			**Laparoscopic Partial Colectomy**	17.3, 17.31, 17.32, 17.33, 17.34, 17.35, 17.36, 17.39	0DTH4ZZ,<br>0DTH8ZZ,<br>0DTK4ZZ,<br>0DTK8ZZ,<br>0DTF4ZZ,<br>0DTF8ZZ,<br>0DTL4ZZ,<br>0DTL8ZZ,<br>0DTLFZZ,<br>0DTM4ZZ,<br>0DTM8ZZ,<br>0DTMFZZ,<br>0DTG4ZZ,<br>0DTG8ZZ,<br>0DTGFZZ,<br>0DTN4ZZ,<br>0DTN8ZZ,<br>0DTNFZZ,<br>0DBE3ZZ,<br>0DBE4ZZ,<br>0DBE8ZZ,<br>0DBH3ZZ,<br>0DBH4ZZ,	44204,<br>44205,<br>44206,<br>44207,<br>44208,

© 2020 Nabi J et al. *JAMA Network Open.*

					0DBH8ZZ, 0DBK3ZZ, 0DBK4ZZ, 0DBK8ZZ, 0DBF3ZZ, 0DBF4ZZ, 0DBF8ZZ, 0DBL3ZZ, 0DBL4ZZ, 0DBL8ZZ, 0DBLFZZ, 0DBM3ZZ, 0DBM4ZZ, 0DBM8ZZ, 0DBMFZZ, 0DBG3ZZ, 0DBG4ZZ, 0DBG8ZZ, 0DBGFZZ, 0DBN3ZZ, 0DBN4ZZ, 0DBN8ZZ, 0DBNFZZ	
			**Robotic Assisted Partial Colectomy**	Above + 17.4, 17.41, 17.42, 17.43, 17.44, 17.45, 17.49	Above + 8E0W4CZ, 8E0W7CZ, 8E0WXCZ, 8E0W0CZ, 8E0W3CZ, 8E0W8CZ	Above + S2900 (HCPCS)

© 2020 Nabi J et al. *JAMA Network Open.*

**Kidney Cancer**	189, 189.0	C64, C64.1, C64.2, C64.9	**Open Radical Nephrectomy**	55.5, 55.52	0TT00ZZ, 0TT10ZZ	50220, 50225, 50230
			**Laparoscopic Radical Nephrectomy**	N/A	0TT04ZZ, 0TT14ZZ	50545, 50546
			**Robotic Assisted Radical Nephrectomy**	Above + 17.4, 17.41, 17.42, 17.43, 17.44, 17.45, 17.49	Above + 8E0W4CZ, 8E0W7CZ, 8E0WXCZ, 8E0W0CZ, 8E0W3CZ, 8E0W8CZ	Above + S2900 (HCPCS)
			**Open Partial Nephrectomy**	55.39, 55.4	0TB00ZZ, 0TB07ZZ, 0TB10ZZ, 0TB17ZZ	50240
			**Laparoscopic Partial Nephrectomy**	N/A	0TB03ZZ, 0TB04ZZ, 0TB08ZZ, 0TB13ZZ, 0TB14ZZ, 0TB18ZZ	50543
			**Robotic Assisted Partial Nephrectomy**	Above + 17.4, 17.41, 17.42, 17.43, 17.44, 17.45, 17.49	Above + 8E0W4CZ, 8E0W7CZ, 8E0WXCZ, 8E0W0CZ, 8E0W3CZ, 8E0W8CZ	Above + S2900 (HCPCS)

© 2020 Nabi J et al. *JAMA Network Open.*
